# Inferring transmission fitness advantage of SARS-CoV-2 variants of concern from wastewater samples using digital PCR, Switzerland, December 2020 through March 2021

**DOI:** 10.2807/1560-7917.ES.2022.27.10.2100806

**Published:** 2022-03-10

**Authors:** Lea Caduff, David Dreifuss, Tobias Schindler, Alexander J Devaux, Pravin Ganesanandamoorthy, Anina Kull, Elyse Stachler, Xavier Fernandez-Cassi, Niko Beerenwinkel, Tamar Kohn, Christoph Ort, Timothy R Julian

**Affiliations:** 1 Eawag, Swiss Federal Institute of Aquatic Science and Technology, Dübendorf, Switzerland; 2 Department of Biosystems Science and Engineering, ETH Zurich, Basel, Switzerland; 3 SIB Swiss Institute of Bioinformatics, Lausanne, Switzerland; 4 Swiss Tropical and Public Health Institute, Basel, Switzerland; 5 University of Basel, Basel, Switzerland; 6 Laboratory of Environmental Chemistry, School of Architecture, Civil and Environmental Engineering, École Polytechnique Fédérale de Lausanne (EPFL), Lausanne, Switzerland

**Keywords:** SARS-CoV-2, B.1.1.7, digital PCR, drop-off assays, transmission fitness

## Abstract

**Background:**

Throughout the COVID-19 pandemic, SARS-CoV-2 genetic variants of concern (VOCs) have repeatedly and independently arisen. VOCs are characterised by increased transmissibility, increased virulence or reduced neutralisation by antibodies obtained from prior infection or vaccination. Tracking the introduction and transmission of VOCs relies on sequencing, typically whole genome sequencing of clinical samples. Wastewater surveillance is increasingly used to track the introduction and spread of SARS-CoV-2 variants through sequencing approaches.

**Aim:**

Here, we adapt and apply a rapid, high-throughput method for detection and quantification of the relative frequency of two deletions characteristic of the Alpha, Beta, and Gamma VOCs in wastewater.

**Methods:**

We developed drop-off RT-dPCR assays and an associated statistical approach implemented in the R package WWdPCR to analyse temporal dynamics of SARS-CoV-2 signature mutations (spike Δ69–70 and ORF1a Δ3675–3677) in wastewater and quantify transmission fitness advantage of the Alpha VOC.

**Results:**

Based on analysis of Zurich wastewater samples, the estimated transmission fitness advantage of SARS-CoV-2 Alpha based on the spike Δ69–70 was 0.34 (95% confidence interval (CI): 0.30–0.39) and based on ORF1a Δ3675–3677 was 0.53 (95% CI: 0.49–0.57), aligning with the transmission fitness advantage of Alpha estimated by clinical sample sequencing in the surrounding canton of 0.49 (95% CI: 0.38–0.61).

**Conclusion:**

Digital PCR assays targeting signature mutations in wastewater offer near real-time monitoring of SARS-CoV-2 VOCs and potentially earlier detection and inference on transmission fitness advantage than clinical sequencing.

## Introduction

Emergence of severe acute respiratory syndrome coronavirus 2 (SARS-CoV-2) genetic variants of concern (VOCs) can impact negatively on efforts to reduce the global disease burden attributable to coronavirus disease (COVID-19). New VOCs emerge when mutations in the viral genome increase transmissibility and/or virulence or reduce neutralisation by antibodies obtained from prior infection or vaccination. Notable examples include Alpha (Phylogenetic Assignment of Named Global Outbreak Lineages (Pangolin) designation lineage B.1.1.7, first described in the United Kingdom in September 2020), Beta (lineage B.1.351, first discovered in South Africa in December 2020) and Gamma (lineage P.1, first discovered in Brazil in January 2021). 

During the timeline of this study (December 2020 through March 2021), Alpha, Beta and Gamma variants spread worldwide, with Alpha overtaking other variants to become the most abundant variant in Europe. The rise in the Alpha variant coincided with a rise in the number of reported infections, motivating Alpha as the target VOC for this study. The VOCs are identified by combinations of co-occurring mutations. Tracking the introduction and transmission of VOCs relies on sequencing, typically whole genome sequencing of clinical samples. Given the need for fast and cheap methods that allow high throughput and are easy to interpret, PCR-based methods targeting signature or defining mutations (typically point mutations or deletions) have become increasingly common [1,2].

Because SARS-CoV-2 RNA is shed in faeces and other bodily fluids [3,4], it is readily detectable in wastewater samples. Surveillance for SARS-CoV-2 in wastewater (wastewater-based epidemiology) is used to inform COVID-19 disease trajectories in catchment areas. Wastewater can also be used to inform the introduction and spread of SARS-CoV-2 variants. To date, sequencing SARS-CoV-2 in wastewater has been applied to detect novel variants in the San Francisco Bay Area [5], detect and track geographical spread of the Alpha variant in Switzerland [6] and track the increased occurrence of Alpha in London [7]. However, in contrast to clinical samples, wastewater samples consist of a mix of SARS-CoV-2 variants representative of the variants circulating in the catchment areas. Therefore, VOCs in wastewater are inferred by tracking combinations of multiple signature or characteristic mutations. Confidence in the detection can be increased by investigating temporal trends and/or tracking signature mutations that co-occur on a single amplicon [6].

Although sequencing SARS-CoV-2 RNA in wastewater is a promising approach to track VOC from hundreds or thousands of individuals in a single sample, there are temporal lags in sample collection and analysis, similar to lags associated with clinical testing. Sequencing and bioinformatics pipelines typically take at least 3 days to yield results after sample RNA is collected. For more rapid identification of VOCs, PCR-based assays have been developed to identify their characteristic mutations [1,2,8]. Examples include assays targeting single-nucleotide polymorphisms, such as N501Y and E484K in the spike protein, or characteristic deletions such as spike Δ69–70 and ORF1a Δ3675–3677. The PCR-based assays, designed for detection of variants in clinical samples, have also been applied successfully for detection in wastewater [9-11]. Using quantitative PCR-based assays (RT-qPCR or RT-dPCR), the proportion of a specific variant within the virus population can be inferred. Proportions are determined using quantitative assays by comparing the quantity of the target gene region characteristic of the variant to the quantity of a conserved region either on a distinct gene (i.e. N gene) or on a region adjacent to the VOC-specific region [9-11]. Although this approach has demonstrated correlation with proportions of variants in clinical samples, bias is introduced by variation in efficiency and sensitivity of the quantification approaches of two different assays.

Here, we developed and assessed methods for inferring the epidemiology of SARS-CoV-2 VOCs from wastewater using digital RT-dPCR assays with non-competitive hydrolysis probes targeting signature mutations of the Alpha, Beta and Gamma VOCs. We aimed to demonstrate that the approach can be used to quantify transmission fitness advantage and provide data similar to those obtained from clinical samples. Overall, our aim was to demonstrate the utility of digital PCR assays targeting signature mutations in wastewater in providing near real-time monitoring of SARS-CoV-2 VOCs and potentially earlier inference on the epidemiology of VOC transmission relative to inferences based on clinical samples.

## Methods

### Overview

We adapted and applied a rapid, high-throughput method for detection and quantification of the frequency of two signature deletion mutations characteristic of the Alpha, Beta and Gamma VOCs in wastewater. We quantify proportions of the variant in a wastewater sample using two non-competitive hydrolysis probes targeting conserved regions on the same amplicon within a single RT-dPCR assay (drop-off RT-dPCR assay). This allowed for simultaneous quantification of wildtype (strain not carrying the mutation of interest) and variants. We applied the assay to raw influent obtained from a wastewater treatment plant in Zurich, Switzerland, over the period December 2020 to March 2021 when Alpha was introduced and spread throughout the region [12]. We further developed a statistical approach to analyse temporal dynamics in drop-off RT-dPCR data to quantify transmission fitness advantage, and compared the results to those obtained from analysis of clinical samples. 

### Drop-off RT-dPCR assays for variant of concern detection in wastewater

We developed a first drop-off RT-dPCR assay using a hydrolysis probe to target the spike Δ69–70 deletion characteristic of the SARS-CoV-2 Alpha variant, and a second assay for ORF1a Δ3675–3,677 characteristic of the Alpha, Beta, and Gamma variants. Although these deletions are characteristic of VOCs, both have been detected in other variants, including non-VOCs [1]. Notably, spike Δ69–70 is associated with increased cell infectivity: in the Alpha variant, it compensates for reduced infectivity caused by immune escape mutations [13,14]. The assay adapts the approach of Vogels et al. in which the hydrolysis probes anneal preferably on amplicons without the deletion and are hereafter referred to as 'deletion probe' [1]. Based on RT-qPCR analysis of a suite of 71 previously sequenced clinical samples, Vogels et al. report that the hydrolysis probes only anneal to the samples without the deletion [1].

**Figure 1:**
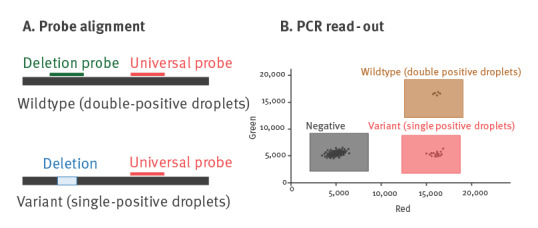
Schematic overview of the SARS-CoV-2 drop-off RT-dPCR assay based on two different probes, a deletion probe and a universal probe

To measure the fraction of VOCs, we adapted the assay by designing additional hydrolysis probes which target conserved regions on the same amplicon as the deletion probes (Table 1). These probes, hereafter referred to as 'universal probes', detect amplicons of all lineages, including those with deletions. Based on the ratio of dPCR droplets with single fluorescence (corresponding to amplicons with a deletion) vs those with double fluorescence (amplicons without the deletion), the proportion of VOCs in the wastewater can be estimated (Figure 1). Further, Alpha variants can be distinguished from Beta and Gamma by comparing dPCR results of the two assays targeting the spike Δ69–70 and ORF1a Δ3675–3677 [1].

**Table 1:** Primer and probe sequences for SARS-CoV-2 drop-off RT-dPCR, including resulting amplicon size and corresponding reference

DNA oligonucleotide name	DNA sequence (5'–3')	Reference
Target: spike ∆69–70 (108 bp amplicon)		
Yale_69–70_F	TCAACTCAGGACTTGTTCTTACCT	[1]
Yale_69–70_R	TGGTAGGACAGGGTTATCAAAC	[1]
*Deletion probe*		
Yale_69–70_Cy5_P	Cy5-TTCCATGCTATACATGTCTCTGGGA-BHQ2	[1], new quencher, fluorophore
*Universal probe*		
LC_69–70_HEX_P	HEX-CCAATGGTACTAAGAG-MGBQ530	This study
Target: ORF1a ∆3675–3677 (128 bp amplicon)		
Yale_ORF1a-del_F	TGCCTGCTAGTTGGGTGATG	[1]
Yale_ORF1a-del_R	TGCTGTCATAAGGATTAGTAACACT	[1]
*Deletion probe*		
Yale_ORF1a-HEX_P	HEX-GTTTGTCTGGTTTTAAGCTAAAAGACTGTG-BHQ1	[1], new quencher, fluorophore
*Universal probe*		
LC_ORF1a-Cy5_P	Cy5-CGTATTATGACATGGTTGGATATGGTTGAT-BHQ2	This study

SARS-CoV-2: severe acute respiratory syndrome coronavirus 2.

SARS-CoV-2: severe acute respiratory syndrome coronavirus 2.

Panel A: The universal probe binds to both the wildtype and the variant, while the deletion probe only binds to the wildtype (no deletion). Panel B: The wildtype appears as double-positive droplets (brown) and the variant as single-positive droplets (red) in the associated dPCR 2D scatter plot.

The drop-off RT-dPCR assays were performed with the Crystal Digital PCR using the Naica System (Stilla Technologies, Villejuif, France) with the qScript XLT 1-Step RT-PCR Kit (QuantaBio, Beverly, Massachusetts, United States (US)). Reactions were prepared as 27 μL (using 5.4 μL template and 21.6 μL mastermix) pre-reactions, of which 25 μL was loaded into Sapphire Chips (Stilla Technologies) for an equivalent of 5 μL template per reaction. Mastermix composition was slightly different depending on the assay (see Supplementary Table S1 for the composition of the reactions). Templates (RNA extracts from wastewater) were diluted 1:2 to maximise the quantification of amplicons per reaction. Although dilution reduces the concentration of template in the sample, it also dilutes out inhibitory substances (the effect of different dilutions on the quantified number of amplicons per reaction is exemplified in Supplementary Figure S1). Any measurement was performed in duplicate. Thermocycler conditions included partitioning droplets (40 °C for 12 min), reverse transcription (55 °C for 30 min) and polymerase activation (95 °C for 1 min), followed by 40 cycles of denaturation (95 °C for 10 s) and annealing/extension (55 °C for 30 s).

### Benchmarking the drop-off RT-dPCR assay

We first tested the performance of the drop-off RT-dPCR assay against RNA extracts of the SARS-CoV-2 Wuhan-Hu-1 lineage (representing the wild-type sample) and of the Alpha variant (representing the VOC sample). Viral strains were obtained from the Spiez Laboratory as reported in Bechtold et al. [15]. RNA extracts from both samples were diluted in molecular grade water to a target concentration of 200 genome copies (gc)/μL. Each hydrolysis probe was first tested in singleplex assays. Then the universal and the deletion probe for each amplicon were combined to yield two duplex assays targeting amplicons inclusive of either spike Δ69–70 or ORF1a Δ3675–3677. To assess matrix effects on assay performance, we tested both duplex assays using samples of wild type or Alpha RNA diluted in RNA extract from wastewater previously tested to contain no SARS-CoV-2 RNA. We did not use RNA from any SARS-CoV-2 Beta or Gamma strains since the method is based on a previous paper by Vogels et al., where a variety of different SARS-CoV-2 genomes have been tested, including Beta and Gamma [1].

The performance of the duplex assays to estimate the ratio of VOCs in a mixed sample was tested in samples containing both wild-type and Alpha RNA with the following proportions of variant: 0.01, 0.02, 0.10, 0.50, 0.90, 0.98 and 0.99, at a total target concentration of 100 gc/μL. Dilutions were performed in molecular grade water.

The limit of quantification (LOQ) was determined by measuring 10 replicates of 25, 30 and 40 gc/reaction using the wild-type RNA. The LOQ was defined as the lowest value where the relative standard deviation is smaller than 25% [16]. For spike Δ69–70, the LOQ was at 40 gc/reaction and for ORF1a Δ3675–3677 at 25 gc/reaction.

### Measuring variants of concern in wastewater

We applied the drop-off RT-dPCR assays to 32 raw influent samples (24 h flow proportional composite) collected from the wastewater treatment plant Werdhölzli (serving ca 471,000 people in Zurich, Switzerland) between 7 December 2020 and 26 March 2021. Samples were stored at 4 °C for on average 2–3 days but some as long as 8 days as they were shipped twice per week from the treatment plant to Eawag (Duebendorf, Switzerland), where they were immediately concentrated and the RNA was extracted. RNA extracts were stored at −80 °C and processed only after assay development (which was 4 months after the first wastewater samples were concentrated and extracted). Throughout this period, a subset of 50 mL wastewater samples were spiked with murine hepatitis virus at concentrations of approximately 10^6^ gc/50 mL to estimate virus recovery as previously described [17].

Sample concentration and RNA extraction followed a previously reported protocol [17,18]. In brief, raw influent (50 mL) was clarified by centrifugation or filtration (0.22 μM filter), then samples were concentrated using ultrafiltration (10 kDa Centricon Plus-70, Millipore, Burlington, Massachusetts, US) resulting in volumes between 150 μL and 300 μL. We then extracted RNA from the full volume of the concentrate using the QiaAmp Viral RNA MiniKit (Qiagen, Hilden, Germany), eluted it in 80 μL and, for samples after 14 January, further purified it using OneStep PCR Inhibitor Removal Kits (Zymo Research, Irvine, California, US) to reduce assay inhibition. Within the scope of the measurements of SARS-CoV-2 N1 gene marker, in which SARS-CoV-2 load is estimated, we measured assay inhibition using synthetic SARS-CoV-2 RNA as a spiked internal control [18]. However, as previously discussed, samples in this study were instead diluted 1:2 to optimise the quantification of target copies per reaction, not to accurately estimate total SARS-CoV-2 concentrations in the sample (see Supplementary Figure S1 for number of target copies quantified in the sample at various dilutions). The dMIQE checklist is available in Supplementary Table S2 [19].

### Data analysis

The statistical methods developed and used for analysing the present data are made available in an R package (R Foundation, Vienna, Austria) 'WWdPCR' (https://github.com/cbg-ethz/WWdPCR). The proportion of amplicons with deletions was calculated for each assay by accounting for the distribution of droplets with no, single and double fluorescence (see Supplement section S1 and Figure S3 for a description of how fluorescence counts were extracted). Statistical methodology to model droplet counts in singleplex dPCR is well developed [20-22]. However, to the best of our knowledge, no proper likelihood model exists for counts generated by duplex drop-off assays. In assays with multiple targets such as duplex assays, positive counts for each probe are typically assumed to be independent [20,23,24]. In a drop-off assay, this assumption is flawed as the number of viral particles positive for the drop-off probe trapped in a partition cannot exceed the number of viral particles positive for the conserved probe. When the relative proportion of variant RNA in the sample is very low (which is common in surveillance of wastewater for SARS-CoV-2) or very high, confidence intervals based on this assumption will be vastly flawed. Here, we developed a likelihood model for the duplex drop-off assay by dropping this problematic assumption. As for singleplex assays [20-22], we assumed that the number 𝑀_𝑖_ of virus RNA genome copies in a droplet 𝑖 is Poisson-distributed with mean 𝜆 = 𝐶𝑣, where 𝐶 is the concentration of SARS-CoV-2 RNA in genome copies per volume and 𝑣 is the droplet volume [20-22]. We further assumed that the number 𝑁_𝑖_ of virus particles without the deletion (double-positive droplets) in a droplet 𝑖 is binomially distributed with sample size 𝑀_𝑖_ such that 𝑟_wt_ corresponds to the relative frequency of the wild type in the viral population.

Assuming independent and identically distributed fluorescence signals of droplets, the counts 𝑋_0_ of double-negative droplets (no virus RNA), 𝑋_1_ of droplets positive for the universal probe but not for the deletion probe (variant RNA), and 𝑋_2_ of double-positive droplets (wild type RNA) follow a multinomial distribution with probabilities of success:


$$p_{0}=e^{-\lambda }$$,

$$p_{1}=e^{-r_{WT}\lambda }-e^{-\lambda }$$,

$$p_{2}={1-e}^{-r_{WT}\lambda }$$

and sample size:


$$n_{tot}{=x}_{0}+x_{1}+x_{3}$$

corresponding to the total number of droplets (see Supplement section S2 for details of derivation). From this likelihood, we find maximum likelihood estimators:


$$\hat{r_{wt}}=\frac{log\left(\frac{x_{0}+x_{1}}{n_{tot}}\right)}{log\left(\frac{x_{0}}{n_{tot}}\right)}$$

and


$$\hat{\lambda }\;=-log\left(\frac{x_{0}}{n_{tot}}\right)$$.

By invariance of the likelihood, the maximum likelihood estimator of the proportion of the mutant allele 𝑟_mut_ in the sample is:


$$\hat{r_{mut}}=1-\hat{r_{wt}}=\frac{log\left(\frac{x_{0}}{x_{0}+x_{1}}\right)}{log\left(\frac{x_{0}}{n_{tot}}\right)}$$.

The temporal increase in the proportion of amplicons with deletions in wastewater, indicative of VOCs, was compared with the temporal increase of the deletions and Alpha in clinical samples in Canton Zurich (region with 1.5 million inhabitants) and Switzerland. In analysing clinical samples from Zurich and Switzerland, we assumed that the proportion of clinical sample genomes that are Alpha or bear a signature mutation were binomially distributed, such that their empirical frequency is the maximum likelihood estimator for their prevalence in the population.

Following Chen et al. [12], we assumed that the relative frequency (𝑡) of a VOC follows a logistic growth with rate *𝑎* and inflection point 𝑡_0_ (see Supplement section S3 for further discussion of model assumptions), such that:


$$r_{VOC}(t)\;=\;\frac{e^{a(t-t_{0})}}{1\;+e^{a(t-t_{0})})}$$

Notably, here and throughout, 𝑡_0_ is defined as days since 7 December 2020, the first day we included in analysis.

As some lineages with no reproductive fitness advantage already present in Zurich can share mutations with the VOC under investigation, we added a background constant prevalence parameter 𝑐 when modelling the relative frequency 𝑟_mut_ (𝑡) of a mutation, such that it follows [25]:


$$r_{mut}(t)\;=c+(1-c)\;\frac{e^{a(t-t_{0})}}{1\;+e^{a(t-t_{0})})}$$

Borrowing from the item response theory literature of psychometrics, we will refer to these models as the two-parameter logistic (2PL) and the three-parametric logistic (3PL) models [25]. We assumed that each observation of the proportion of a mutation is an independent observation. Therefore, a best fit can be found by maximising the joint (log-)likelihood of either the binomial counts in clinical data or the multinomial counts in wastewater data. The L-BFGS-B algorithm implemented in the R v4.0.5 package 'stats' was used for the optimisation and estimation of the Fisher information matrix. We then computed Wald confidence intervals for the parameter estimates using the standard errors from quasi-binomial and quasi-multinomial distributions to account for over-/underdispersion [26,27]. The estimates of the growth rate parameter *𝑎* were transformed along with their confidence intervals into transmission fitness advantage 𝑓_d_, assuming the discrete-time logistic growth model of Chen et al. and a generation time 𝑔 = 4.8 days, such that 𝑓_d_ = 𝑒^ag^ − 1 [12]. Wald confidence bands for the fitted values were calculated using the Delta method [27] for the logit-transformed fitted values and then back-transformed to the original scale, so as to ensure that they were restricted to the interval [0,1]. Approximate confidence bands were also calculated on the logit scale and then back-transformed. For each time series, both 3PL and 2PL were fit to the data and then compared by way of a likelihood ratio test based on quasi-likelihood [26], and the 3PL model was retained only if p < 0.05. The assumption of constant overdispersion of the quasi-likelihood models was checked graphically (Supplementary Figure S5).

### Ethical statement 

Ethical approval was not needed for this study. SARS-CoV-2 RNA detected from wastewater in a large catchment area (471,000 people) provides anonymised data. Clinical data used for comparison were obtained from the publicly available database accessible through CovSpectrum (cov-spectrum.org) [28].

## Results

### Benchmarking the drop-off RT-dPCR assay

Both drop-off assays targeting the spike Δ69–70 and the ORF1a Δ3675–3677 performed as expected in singleplex and duplex assays. Specifically, the universal probe was detected in singleplex and duplex assays when using RNA from both the Alpha VOC sample and the wild-type strain. Similarly, the probe targeting only the wild-type strain (deletion probe) was only detected in the wild-type strain. Notably, when the assays were applied to the wild-type RNA, the universal probe and the deletion probe reported nearly identical concentrations. In the spike Δ69–70 assay, the concentrations measured were 162.3 gc/μL for the universal probe and 162.8 gc/μL for the deletion probe. For the ORF1a Δ3675–3677 assay, the concentration measured was 155.2 gc/μL for both probes. When testing the ratio of the Alpha RNA to the wild-type strain, both duplex assays performed as expected. The expected ratio and the measured ratio were linearly correlated (see Supplementary Figure S6). The exception was one replicate of the sample in the spike Δ69–70 assay with a variant-to-wild type RNA ratio of 1:50, in which the variant RNA was not detected.

The assay performance was the same in wastewater matrix, with both the universal and the deletion probes detectable in the wild-type strain RNA and only the universal probe detectable in the VOC Alpha RNA. Concentrations were nearly identical for the wild-type RNA for the universal (43.3 gc/μL) and deletion (42.9 gc/μL) probes in the spike Δ69–70 assay and for the universal (42.2 gc/μL) and deletion (42.2 gc/μL) probes in the ORF1a Δ3675–3677 assay.

### Measuring variants of concern in Zurich wastewater

**Figure 2:**
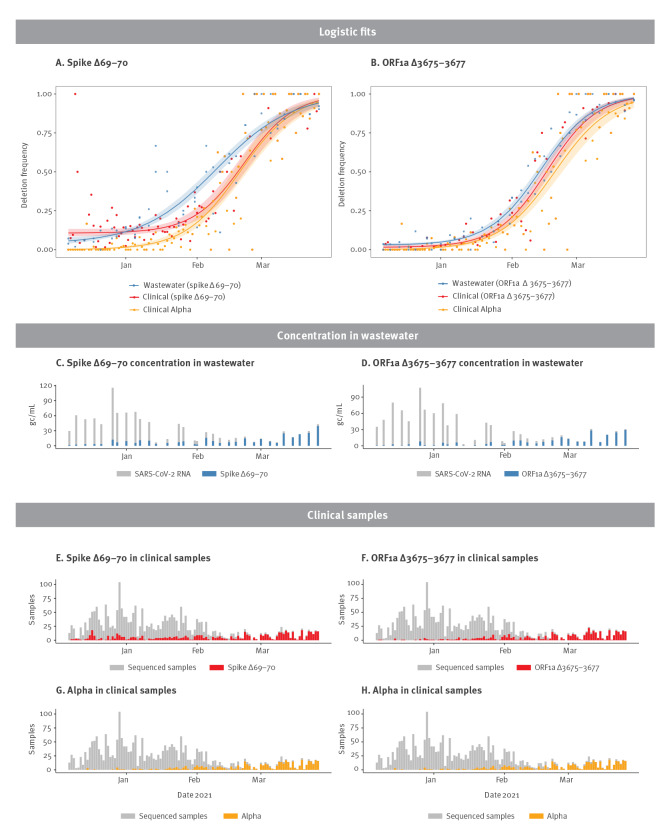
Proportion of SARS-CoV-2 deletion mutants in wastewater and clinical samples and proportion of Alpha lineage in clinical samples, Zurich, Switzerland, December 2020–March 2021 (n = 32)

In the wastewater samples, concentrations reported by the universal probes (per mL wastewater) ranged from 2.0 gc/mL to 60.6 gc/mL for the spike Δ69–70 assay and from 0.50 gc/mL to 65.0 gc/mL for the ORF1a Δ3675–3677 assay (Figure 2). Concentrations of amplicons with deletions, estimated based on the number of single-positive droplets, increased over time and ranged from 0.6 gc/mL to 20.4 gc/mL for the spike Δ69–70 assay and from undetectable to 15.8 gc/mL for the ORF1a Δ3675–3677 assay. Notably, spike Δ69–70 was detectable throughout the analysis period, probably due to carriage of the deletion in non-Alpha clinical samples. Recovery of MHV was 0.09% (standard deviation: 0.06%), in line with previous results [17].

SARS-CoV-2: severe acute respiratory syndrome coronavirus 2.

Panels A and B: Fitted curves: three-parametric logistic fits for the deletions in wastewater and clinical data and two-parametric logistic fit for the Alpha clinical data; shaded areas: 95% confidence bands. Panels C and D: concentration of SARS-CoV-2. Panels E–H: Number of Zurich sequenced clinical samples.

Quantification of the proportion of deletions in wastewater aligned with the proportion of deletions detected in clinical samples from both Zurich (Figure 2, see Supplementary Figure S7 for the same fits with prediction bands) and Switzerland (see Supplementary Figure S4 for the comparison of wastewater samples with clinical samples throughout Switzerland and see Figure S8 for fits with prediction bands). Notably, both deletions were detected early in wastewater, probably reflecting the presence of the deletions in variants other than Alpha, Beta and Gamma. Indeed, 3.4% of 8,709 clinical samples contained spike Δ69–70 and 0.06% contained ORF1a Δ3675–3677 in Switzerland before 24 December 2020 [6].

### Estimating Alpha growth rate and transmission fitness in wastewater

Accounting for these background prevalence rates in the logistic growth model, estimates for the growth rate 𝑎, time to maximum growth 𝑡_0_, and transmission fitness advantage 𝑓_d_ were generally consistent with estimates obtained from clinical samples across Switzerland (Table 2). Specifically, the growth rate estimate from wastewater was 0.06 (95% confidence interval (CI): 0.06–0.07) for spike Δ69–70 compared with an estimate of 0.09 (95% CI: 0.07–0.11) from Zurich clinical samples. Similarly, the growth rate estimate was 0.09 (95% CI: 0.08–0.09) for ORF1a Δ3675–3677 in wastewater compared with 0.09 (95% CI: 0.08–0.10) from Zurich clinical samples. Converting growth rates to transmission fitness advantage further highlighted the similarities, with wastewater estimates for spike Δ69–70 of 0.34 (95% CI: 0.30– 0.39) and for ORF1a Δ3675–3677 of 0.53 (95% CI: 0.39–0.73). These estimates of transmission fitness advantage are similar to those based on clinical samples, using the fractions of both deletions and using the fraction of Alpha variant cases. Table 2 shows that confidence intervals for the parameters of epidemiological models fitted on both types of data are for the greater part overlapping.

**Table 2:** Logistic growth model parameter estimates for the prevalence of SARS-CoV-2 spike Δ69–70 and ORF1a Δ3675–3677 in wastewater (n = 32), Swiss clinical (n = 8,877) and Zurich clinical samples (n = 2,497), and of Alpha variants in Swiss and Zurich clinical data, Switzerland, 7 December 2020–26 March 2021

Target	Sample type	Growth rate (*𝑎)*	Time to maximum growth *(*𝑡_0_ *)*	Background prevalenc*e (*𝑐*)*	Transmission fitness advantage *(*𝑓_d_ *)*
Alpha	Switzerland (clinical)	0.07 (0.07–0.08)	66.1 (64.4**–**67.8) 11 Feb (9–13)	0 (fixed)	0.42 (0.38–0.46)
	Zurich (clinical)	0.08 (0.07–0.1)	74.6 (70.0–79.2) 20 Feb (15–24)	0 (fixed)	0.49 (0.38–0.61)
Spike Δ69–70	Wastewater	0.06 (0.06–0.07)	64.5 (62.0–67.1) 10 Feb (7–12)	0.04 (0.01–0.06)	0.34 (0.30–0.39)
	Switzerland (clinical)	0.07 (0.06–0.08)	65.5 (63.5–67.6) 11 Feb (8–12)	0.07 (0.04–0.09)	0.41 (0.36–0.46)
	Zurich (clinical)	0.09 (0.07–0.11)	76.3 (71.8–80.9) 21 Feb (17–25)	0.11 (0.07–0.14)	0.55 (0.39–0.73)
ORF1a Δ3675–3677	Wastewater	0.09 (0.08–0.09)	68.5 (67.4–69.6) 14 Feb (12–14)	0.03 (0.02–0.04)	0.53 (0.49–0.57)
	Switzerland (clinical)	0.09 (0.08–0.11)	68.7 (66.1–71.2) 14 Feb (11–13)	0.03 (0–0.05)	0.56 (0.45–0.67)
	Zurich (clinical)	0.09 (0.08–0.1)	71.3 (68.8–73.7) 17 Feb (15–19)	0.01 (0–0.03)	0.55 (0.46–0.65)

SARS-CoV-2: severe acute respiratory syndrome coronavirus 2.

Logistic model parameter estimates for the prevalence of spike Δ69–70 and ORF1a Δ3675–3677 in wastewater data, Swiss clinical data and Zurich clinical data are from a three-parametric logistic model (3PL) to account for possible background prevalence of the mutations. Logistic model parameter estimates for the prevalence of Alpha variants in Swiss and Zurich clinical data are from a two-parametric logistic model (2PL). Values are maximum likelihood estimates and Wald 95% confidence intervals of the growth rate 𝑎, midpoint 𝑡_0_ (in days after 7 December 2020 and corresponding dates) and (in the case of 3PL) background prevalence 𝑐. Values for the rate parameter 𝑎 are also shown transformed (along with their confidence intervals) into an estimate of the transmission fitness advantage 𝑓_𝑑_ assuming the discrete-time growth model found in Chen et al. [12]. 3PL models are shown only when the inclusion of a third parameter (background prevalence) was statistically significant (Supplementary Table S2).

Estimates of time to maximum growth estimates were also generally similar when based on clinical or wastewater samples, falling within a range of less than 2 weeks in mid-February (10–21 February 2021). Here, estimates from Zurich clinical samples lagged behind both wastewater and Swiss clinical samples for both assays, with greater deviation for the spike Δ69–70 deletion (Table 2). Similarly, the estimated background prevalences *𝑐* of the mutations were in general consistent between models based on the wastewater data and on the clinical data. The inclusion of a background prevalence parameter statistically significantly improved the fit in all models based on prevalence of deletions, but not in any model based on the prevalence of the Alpha variant in clinical data (Supplementary Table S2 provides additional detail on model parameter estimates for logistic growth models based on wastewater and clinical samples using both two parametric and three parametric fits).

## Discussion

Drop-off RT-dPCR assays for targeted detection of signature mutations in wastewater with a catchment of ca 471,000 inhabitants show temporal trends consistent with sequencing data from clinical samples from the same canton (1.5 million inhabitants). By incorporating non-competitive probes on a single amplicon, proportions of the mutation in the virus population can be directly estimated with a single assay that is not biased by inter-assay variation in quantification. The analysis of wastewater in this study included 32 samples, which contrasts with 2,497 clinical samples collected in Canton Zurich and sequenced during the period of study (7 December 2020 to 26 March 2021). The demonstrated approach provides a method of tracking signature mutations in a community at lower cost and with faster turnaround than clinical or wastewater sequencing, and can be adapted to screen for other signature mutations of other VOCs.

Temporal trends in signature mutations (here, ORF1a Δ3675–3677 and spike Δ69–70) of VOCs tracked in wastewater using dPCR can rapidly inform variant transmissibility. Point estimates for advantages in growth rate and transmission fitness derived from wastewater were generally consistent with point estimates derived from clinical samples: the 95% confidence intervals overlapped as shown in the confidence intervals of the parameter estimates. Because dPCR sample analysis is faster than whole-genome sequencing, the workflow presented here allows insights into transmissibility of novel variants once signature mutations are identified.

Notably, we found that the proportion of variants in wastewater increased earlier than in clinical samples in Zurich. In wastewater samples, the maximum growth rate of ORF1a Δ3675–3677 occurred 2.8 days earlier than in clinical samples, and the maximum growth rate of spike Δ69–70 occurred 11.8 days earlier than in clinical samples. There are a number of possible explanations for the discrepancy in dates between clinical samples and wastewater samples. Firstly, the dates of the two types of samples represent distinct events, with the wastewater samples representing the 24 h period when the wastewater sample was collected and the dates of the clinical samples representing the date a person visits a testing facility. The dynamics of clinical testing may not align with the dynamics of shedding into wastewater. Previous work comparing temporal trends in cases with temporal trends in Zurich wastewater suggests this may only partially contribute to the discrepancy. Wastewater signals for SARS-CoV-2 were observed to be at most 1 day earlier than clinical case data, based on the average delay between infection and date of testing in Zurich (8.1 days) compared with the estimated delay between infection and shedding of SARS-CoV-2 RNA in wastewater (7–11 days) [18]. A second explanation may be that shedding (i.e. concentration, duration) of SARS-CoV-2 RNA into the wastewater may vary by variant. In clinical samples of SARS-CoV-2 RNA measurements from nose/throat samples, people infected with the Alpha variant shed substantially higher viral concentrations than people infected with non-Alpha variants [29]. If more Alpha SARS-CoV-2 RNA is shed into wastewater per person infected as compared with non-Alpha variants, then the proportion of variant amplicons in wastewater would be skewed towards Alpha and would not be an accurate estimate of the proportion of infections with the Alpha variant. Although there is a variant-specific impact on shedding observed from nose/throat samples, it is largely unknown if there is also a variant-specific impact on faecal shedding. Variants may influence not only the magnitude of enteric shedding, but also the proportion of infected people with enteric shedding. Theoretically, wastewater-based estimates on the frequency of VOCs in the population could be corrected to account for variant-specific differences in enteric shedding, but this would require reliable estimates of enteric shedding load profiles for each variant, which are currently lacking.

We applied the drop-off RT-dPCR assay here for signature mutations of SARS-CoV-2 Alpha, Gamma and Beta, but the assay is readily adaptable to screen for other signature mutations beyond those presented here. Targeting signature deletions allows for the design of specific probes annealing to the deleted region to distinguish target variants from other lineages. However, probes providing specificity to other mutations, including single nucleotide polymorphisms, could be targeted by future drop-off assays. Examples include mutations that may influence not only transmissibility but also virulence and/or vaccine-induced antibody escape [13].

The methodology has some limitations. Notably, wastewater-based estimation of the proportion of signature mutations in communities requires sufficient concentrations of SARS-CoV-2 RNA such that relative proportions of target mutations can be quantified. For example, lags in sample processing may have decreased concentrations of SARS-CoV-2 RNA in the samples [17]. Methodological improvements to increase the quantity of SARS-CoV-2 RNA extracted from wastewater are needed to apply this method in scenarios of low viral RNA concentrations in wastewater (corresponding to few clinical cases in the catchment) [30]. Although concentrations of the deletion amplicons in the wastewater often are below the limit of quantification (defined as the lowest concentration with a coefficient of variation ≤ 25%), transmission fitness estimates remain aligned with estimates from clinical samples. This finding suggests that low SARS-CoV-2 RNA concentrations in wastewater may nevertheless be sufficient to infer epidemiological data when analysed as a time series. In addition, the methodology assumes that the universal probe targets a region of the amplicon conserved across all strains and therefore is an accurate indicator of the total number of SARS-CoV-2 amplicons in the sample. Strain-specific variation in the efficiency of amplification of the universal probe, for example because of mutations or differential sample inhibition [31], would result in inaccurate estimation of the total number of amplicons and corresponding inaccuracy of the proportion of the target variant. To address this, continual monitoring of clinical sample sequences for mutations in the target regions of the primers and both universal and wild-type probes is recommended.

## Conclusion

Drop-off RT-dPCR assays provide an opportunity for rapid detection and quantitative estimates of VOCs in communities, offering epidemiological insights into their introduction and transmission. Such assays offer complementary tools to sequencing analyses of VOCs which may be useful for large-scale programmes in wastewater-based epidemiology of SARS-CoV-2 such as the US National Sewer Surveillance System and the European Union Sewer Sentinel Surveillance Network.

## Supplementary Data

1222000 Supplement
